# Computational Approaches for the Discovery of Human Proteasome Inhibitors: An Overview

**DOI:** 10.3390/molecules21070927

**Published:** 2016-07-16

**Authors:** Romina A. Guedes, Patrícia Serra, Jorge A. R. Salvador, Rita C. Guedes

**Affiliations:** 1 iMed.ULisboa, Research Institute for Medicines, Faculdade de Farmácia da Universidade de Lisboa, Av. Prof. Gama Pinto, 1649-003 Lisboa, Portugal; rpaguedes@gmail.com (R.A.G.); patriciafaserra@gmail.com (P.S.); 2 Laboratório de Química Farmacêutica, Faculdade de Farmácia da Universidade de Coimbra, Pólo das Ciências da Saúde-Azinhaga de Santa Comba, 3000-548 Coimbra, Portugal; salvador@ci.uc.pt; 3 Center for Neurosciences and Cell Biology, Universidade de Coimbra, Rua Larga-Faculdade de Medicina, Pólo I, 3004-504 Coimbra, Portugal

**Keywords:** cancer, proteasome inhibitors, computer-aided drug design, virtual screening, molecular docking, pharmacophore model

## Abstract

Proteasome emerged as an important target in recent pharmacological research due to its pivotal role in degrading proteins in the cytoplasm and nucleus of eukaryotic cells, regulating a wide variety of cellular pathways, including cell growth and proliferation, apoptosis, DNA repair, transcription, immune response, and signaling processes. The last two decades witnessed intensive efforts to discover 20S proteasome inhibitors with significant chemical diversity and efficacy. To date, the US FDA approved to market three proteasome inhibitors: bortezomib, carfilzomib, and ixazomib. However new, safer and more efficient drugs are still required. Computer-aided drug discovery has long being used in drug discovery campaigns targeting the human proteasome. The aim of this review is to illustrate selected in silico methods like homology modeling, molecular docking, pharmacophore modeling, virtual screening, and combined methods that have been used in proteasome inhibitors discovery. Applications of these methods to proteasome inhibitors discovery will also be presented and discussed to raise improvements in this particular field.

## 1. Introduction

### 1.1. The Proteasome

The existence of a highly regulated turnover of cellular proteins contributes to cellular homeostasis, a delicate balance between protein synthesis and protein degradation mechanisms [1]. In this way, denatured proteins, damaged proteins (due to, for example, oxidative stress) or proteins that are no longer needed, are recognized and removed through proteolytic degradation, catalyzed by proteases that cleave peptide bonds [2,3,4,5,6].

In eukaryotic cells, two main pathways are responsible for intracellular protein degradation: the lysosomal pathway and the ubiquitin-proteasome pathway (UPP), also known as the ubiquitin-proteasome system (UPS). The UPS plays a pivotal role in degrading proteins in the cytoplasm and nucleus of eukaryotic cells, regulating a wide variety of cellular pathways, including cell growth and proliferation, apoptosis, DNA repair, transcription, immune response, and signaling processes via degradation of cellular key players, such as cyclins, or tumor suppressors (e.g., p53) [4,5,7,8]. Proteins modified with an ubiquitin chain bind to ubiquitin receptors that link them to the 26S proteasome, which degrades ubiquitinated proteins and recycles the ubiquitin for reuse [1,2,9]. The 26S proteasome plays an important role in ATP dependent protein degradation [4,10,11].

The therapeutic potential of intervention in the UPS has been already demonstrated by the development of proteasome inhibitors as a rational therapeutic approach in several diseases such as cancer [12], autoimmune diseases (myasthenia gravis, multiple sclerosis, lupus) [13,14,15], inflammatory pathologies (asthma, psoriasis) [16,17], organ transplant [18], infective diseases (malaria) [19,20,21,22], among others that have been already demonstrated and need to be explored [23].

**Figure 1:**
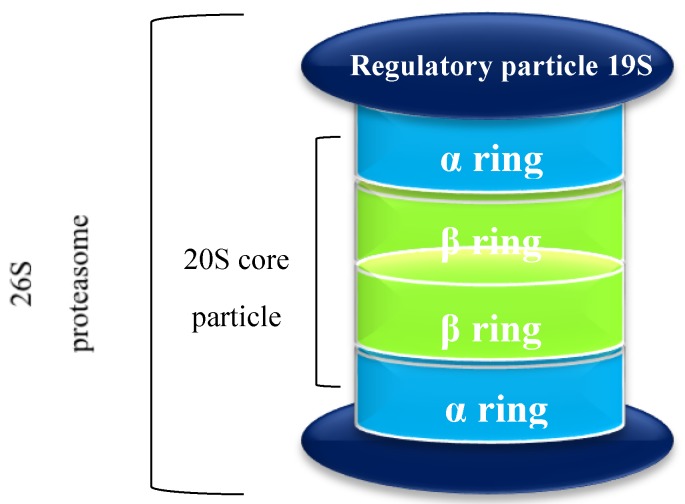
The 26S proteasome schematic representation.

The 26S proteasome has about 2500 kiloDaltons (kDa) of molecular mass and is composed of a 20S core particle and two 19S regulatory particles (Figure 1). The 20S core particle is the key component of the UPS, containing several active centers (catalytic subunits) to degrade unneeded or damaged proteins, unfold protein-substrates and stimulate proteolytic activity [24,25,26,27]. However, beyond its constitutive form, the proteasome can also exist as immunoproteasome, hybrid proteasome, and thymoproteasome [28,29,30,31]. Structurally, yeast and mammal proteasomes present a cylindrical shape of about 160 Å in length and 120 Å in diameter [2,32]. This structure is composed of four rings (two α rings and two β rings organized as α-β-β-α) and each ring is composed of seven different subunits (α1-α7 and β1-β7) [33,34,35,36]. 

From now on, when we refer to “proteasome” it will be related to the 20S core particle.

The 20S proteasome has a central pore whose structure can be subdivided into two fore chambers (located in the area where the α ring contacts with the β ring which is close to it) and one main chamber (which corresponds to the central pore area formed by the contact of the two β rings), being the catalytic core of the proteasome located at the inner rings (the β rings) [2]. However, α rings can change the proteasome activity and specificity since they are responsible for substrate recognition and regulation of substrate access to the inner proteolytic chamber. The substrates entrance in the proteasome can be modulated through the connection of a regulatory particle (such as 19S, 11S or PA200) to the α rings [2,10,11,37]. The main function of β subunits is proteolytic activity, which leads to peptides composed of 3–30 amino acids, predominantly fragments of 6- and 8-amino acids. Many of these resulting peptides are then degraded into individual amino acids through aminopeptidases activity [33,38,39].

In yeast and mammals, each of the three proteolytic subunits presents differences when binding to substrates and activity performed: β1 subunit presents “caspase-like” (C-L) or “post acidic” (PA) activity and cleaves peptide bonds after acidic amino acids; β2 subunit has “trypsin-like” (T-L) activity, and cleaves peptide after basic amino acids; β5 subunit has “chymotrypsin-like” (CT-L) activity and acts after neutral amino acids [40,41,42]. The β subunits enzymatic activity is associated with the Thr1 terminal N-residues, in which the γ hydroxyl group (Thr1Oγ) acts as a nucleophile in the hydrolysis of the peptide bond. Due to this, the proteasome is classified as a member of the N-terminal nucleophilic (Ntn) hydrolases superfamily [43,44,45].

**Figure 2:**
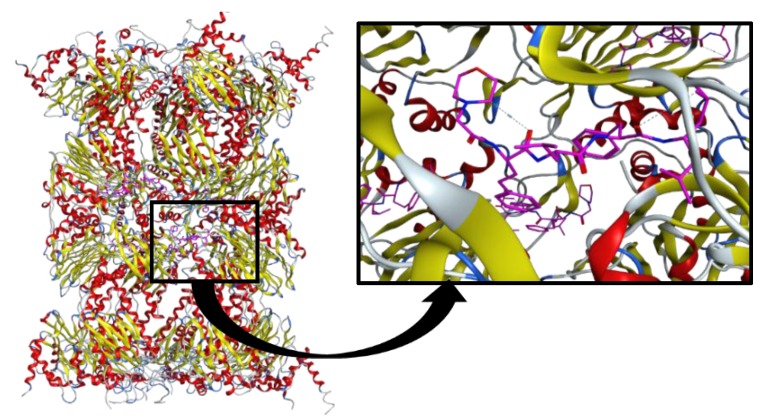
Crystallographic structure of the human constitutive 20S proteasome complexed with carfilzomib (pink) at 2.6 Å resolution (PDB ID: 4R67).

The most relevant active site amino acids are threonine 1 (Thr1), aspartate 17 (Asp17), lysine 33 (Lys33), serine 129 (Ser129), aspartate 166 (Asp166), and serine 169 (Ser169). Thr1, Asp17, and Lys33 are the most important residues in the proteolytic mechanism. The other residues (Ser129, Asp166, and Ser169) contribute not only for catalysis, but also for the active site structural integrity [28,46]. The 3D structure of the proteasome of several organisms has long been investigated through X-ray crystallography and it has been shown that they share the same basic architecture [37]. However, only recently, Harshbarger et al. [47] determined for the first time the crystallographic structure of the human constitutive 20S proteasome free and complexed with the well-known proteasome inhibitor carfilzomib (Krypolis^TM^, Thousand Oaks, CA, USA) at 2.9 Å and 2.6 Å resolution, respectively (Figure 2).

### 1.2. Proteasome Inhibitors

Based on a classification proposed by Kisselev et al. [23], inhibitors of the 20S proteasome can be divided into two main groups based on whether or not they form a covalent bond with the active site Thr1 [23].

Covalent inhibitors usually consist of an electrophilic trap that reacts with the active site Thr1. Based on the nature of electrophilic traps employed for these purposes, eight major classes of proteasome inhibitors can be distinguished: peptide aldheydes, peptide boronates, peptide α′,β′-epoxyketones, peptide ketoaldehydes, β-lactones, peptide vinyl sulfones, syrbactins, and oxatiazol-2-ones [23].

Noncovalent proteasome inhibitors are devoid of a reactive function prone to a nucleophilic attack, which could be an advantage in improved selectivity, less excessive reactivity and instability which are often associated with side effects [48]. The classes of noncovalent proteasome inhibitors include cyclic peptides, noncyclic peptides, peptide isosteres, nonpeptide inhibitors, and hydroxyureas. In terms of reversibility of the binding mode, all noncovalent inhibitors are reversible and so are some covalent inhibitors (e.g., aldehydes and, to some degree, boronates) [23].

Besides the classes of the two main groups, nonspecific proteasome inhibitors, allosteric inhibitors, and site specific inhibitors should also be considered [23].

**Figure 3:**
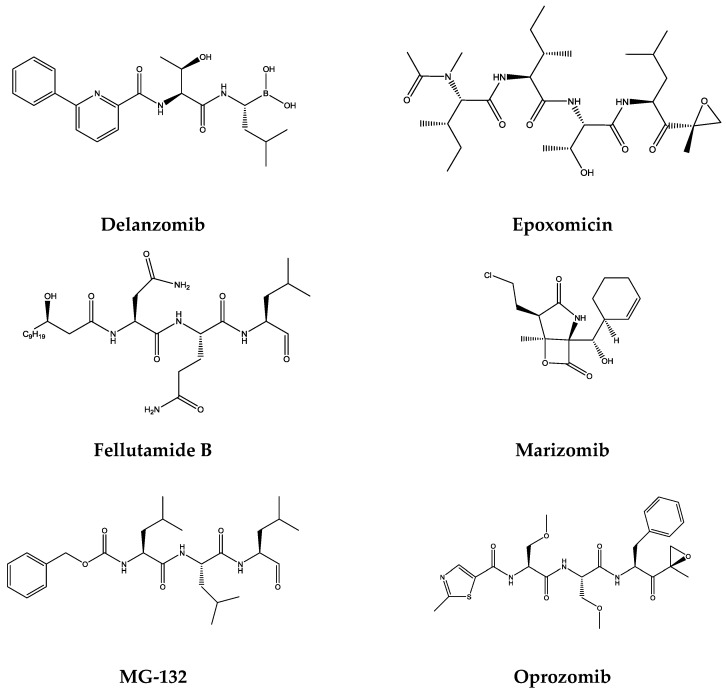
Molecular structures of some proteasome inhibitors.

In Figure 3 the molecular structure and activity of some proteasome inhibitors from different representative classes are displayed.

In Table 1 it is possible to find the inhibitory activity of some relevant proteasome inhibitors (IC_50_ values) in the respective catalytic subunit and the respective structure. 

**Figure 4:**
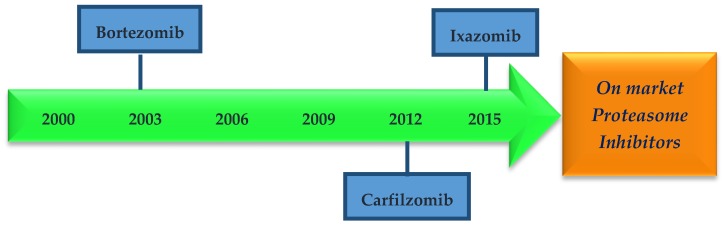
Timeline of on market proteasome inhibitors approved by the FDA.

**Figure 5:**
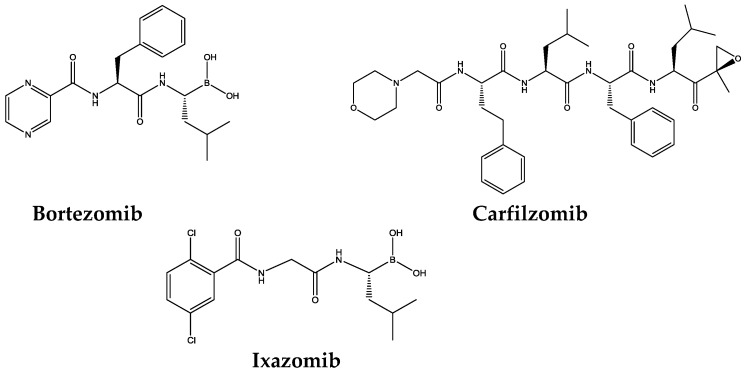
Molecular structure of the three proteasome inhibitors currently on the market.

The extensive research in the field of proteasome inhibitors has led to three drugs on the market: bortezomib, carfilzomib, and ixazomib (Figure 4 and Figure 5) and several others in clinics and clinical trials.

In 2003 (Figure 4), the U.S. Food and Drug Administration (FDA) approved the first proteasome inhibitor for human use, the dipeptidyl boronic acid derivative bortezomib (Velcade^TM^), an intravenous administration used for the treatment of refractory multiple myeloma and mantle cell lymphoma [23,36,56,57,58,59,60]. Bortezomib was developed by Myogenics, which was renamed Proscript and then acquired by Leukosite, being the last company purchased by Millenium Pharmaceuticals, Inc. Johnson & Johnson Pharmaceutical Research & Development is the current company that markets bortezomib [36,61].

Later, in 2012, the FDA granted accelerated approval for the intravenous second-generation proteasome inhibitor carfilzomib (Kyprolis^TM^, Onyx Pharmaceuticals, Inc. an Amgen Inc. subsidiary, Thousand Oaks, CA, USA) for the treatment of patients with multiple myeloma who have received at least two prior therapies, including bortezomib and an immunomodulatory agent, and who revealed disease progression on/or within 60 days of the completion of the last therapy [56,62,63]. 

Very recently (November 20 2015), the U.S. Food and Drug Administration approved ixazomib (Ninlaro^TM^ from Millennium Pharmaceuticals, now Takeda Pharmaceutical Company Limited, Chuo-ku, Osaka, Japan) in combination with lenalidomide and dexamethasone for the treatment of patients with multiple myeloma who have received at least one prior therapy [64,65]. Ixazomib is the first oral proteasome inhibitor on the market [12,65]. 

Despite the efforts of academia and the pharmaceutical industry to develop proteasome inhibitors, effective and safe drugs are still missing. Thus, we believe that the discovery of new drugs requires more basic research. By using all the tools at our disposal we aim to accelerate this process and successfully identify effective therapeutic drugs.

## 2. Computer-Aided Drug Design

The drug development pipeline takes, on average, about 10 to 15 years consisting of a huge financial investment to find a new drug. In an attempt to speed up this process and decreasing simultaneously financial efforts, academia and the pharmaceutical industry are making use in this process of all available strategies, including computational-aided drug design [66,67,68]. 

Computer-Aided Drug Design (CADD) consists in the use of computational methodologies and tools to identify, design and optimize biologically active compounds, that can be synergistically integrated with other related medicinal chemistry fields, for example, synthetic chemistry, as well as biology and pharmacology in order to speed up the drug discovery process [69,70,71,72].

**Figure 6:**
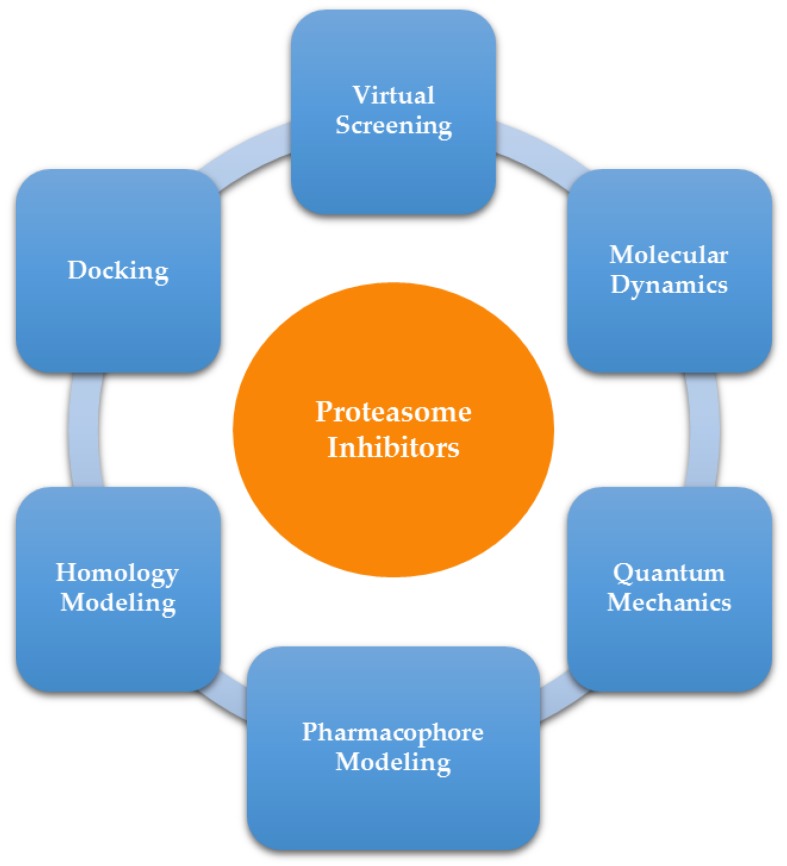
Some Computer-Aided Drug Design (CADD) methodologies used in the discovery and study of proteasome inhibitors.

In this review, an outline of the application of selected CADD methodologies for the discovery and development of proteasome inhibitors, namely molecular dynamics, quantum mechanics, homology modeling, pharmacophore modeling, docking, and virtual screening is described (Figure 6) and illustrative examples from the literature are presented.

### 2.1. Homology Modeling

Rational structure-based drug design is founded on the detailed knowledge of biological systems. A key issue in the use of this kind of methodology relies on the availability of high-resolution structural information. Nowadays, it is normally simple to access this kind of information for a larger number of biological targets due to improved structural characterization methods (X-ray crystallography, multidimensional NMR spectroscopy, among others). However, often, the tridimensional structure that we want to study is missing. Difficulties associated with the determination of a crystallographic structure depend on a variety of reasons such as a difficult process of overexpression, purification and crystallization associated with some biological structures due, for example, to a high mobility, loss of some fraction of the protein during the described process or combination with other structures [73]. The structural characterization of eukaryotic heteromeric proteins is particularly difficult, predominantly due to their size, architecture, instability, and association with other cellular proteins, which makes purification and crystallization very tricky. However, the tight interactions established between 20S proteasome subunits and their condensed fold facilitates in some organisms their structural characterization [74]. Unfortunately, in mammalian 20S proteasomes the purification and crystallization processes are not so simple, mainly due to the presence of interferon γ that causes the replacement of certain proteasome β subunits by others, namely β1 by β1i, β2 by β2i and β5 by β5i [75], leading to the presence of various proteasome subpopulations [76,77].

Until November 2015, the crystallographic structure of human 20S proteasome was unknown and all previous computational structure-based design studies of proteasome inhibitors were based on the structure of the proteasome of other organisms, sharing a large similarity to the human one, such as *Bos taurus*, *Mus musculus*, and *Saccharomyces cerevisiae* (Table 2), which were directly used or were the basis (template) for the generation of human proteasome homology models. 

In the case of a lack of crystallographic structure, partial or complete, homology modeling is the perfect tool to obtain a structure model [73,78,79,80]. Homology modeling’s main goal is to make a prediction of the tridimensional structure of a protein based on the fact that proteins with a homologue amino acid sequence share similar structures (templates) [78,81].

The development of a homology model is not a one step process and it can require several steps until a suitable model is obtained. The construction of a starting model is followed by an optimization and/or refinement phase through energy minimization using a molecular mechanics force field. The model can then be submitted to other techniques such as molecular dynamics simulations to improve model quality [78]. The reliability of a model can be assessed based on the percent identity between amino acid sequence of the protein of interest and the template as well as on the quality of the alignment. It is important to preserve a percentage of identity and sequence similarity above 30% (or preferentially 50%) to use a certain structure as template [73]. The choice for creating models must be supported between sequence-related target structures and functionally-related target structures. This fact is mandatory to understand how sequence, function, and structure are interrelated [80]. Validation methodologies can be divided into two different procedures: (1) Stereochemical analyses of the model, focusing on symmetry, geometry, chirality, torsion angles, ligation angles and their distance; (2) Analyses of the correspondence between the sequence and the template (fitness) and attribution of a score to each residue correctly positioned [78]. Using a unique template or combination of several templates, and information of key points in the structure which are important for a specific process, are essential factors that can lead to the strongest model [73]. 

Homology modeling is an important methodology that can have numerous applications, namely in virtual screening, structure-based drug development approaches and elucidation of the mechanism of a specific process [73]. Table 2 includes some examples of available crystallographic structures of proteasome from *Homo sapiens* and other organisms and also displays the percentage of identity between human and bovine, murine and yeast amino acid sequences for the three catalytic subunits calculated through the algorithm “Basic Local Alignment Search Tool” [82]. Accessing BLAST in UniProt website [83] and complementing the analysis with Molecular Operating Environment (MOE) [84] software, it is possible to conclude that *Mus musculus* and *Bos taurus* are the species whose amino acid sequences have a higher percent identity when compared to the corresponding structures of the human proteasome: e.g., the murine β5c subunit has a percent identity of 95%, the bovine β5c subunit has a percent identity of 96% and, in the yeast proteasome, the percent identity of the β5c subunit is 67%.

In 2004, Furet et al. [85] constructed a homology model using the crystallographic structure of the yeast proteasome of the β-subunits that constitute the CT-L proteolytic site to develop a new class of potent noncovalent 20S proteasome inhibitors, resulting in an improved family of compounds with better results at the cellular level. The homology model was created using the homology module of Insight II [85].

With the aim of identify the determinants of subunit selectivity, in 2014 Loizidou et al. [86] performed a computational study of the subunit specific interactions of the proteasome inhibitors argyrin A and F. To achieve this purpose, homology models of the proteasome active sites (C-L, T-L, CT-L) were developed starting from the crystallographic structure of the yeast proteasome (PDB ID: 2F16) and using the *Homo sapiens* amino acid sequences obtained from UniProt. The alignment of the sequences was performed with the multiple alignment mode of ClustalX 2.1 (Conway Institute UCD, Dublin, Ireland). Subsequently, molecular docking calculations were performed to evaluate the key protein-ligand interactions [86].

### 2.2. Pharmacophore Modeling

The first definition of pharmacophore was suggested by Paul Ehrlich in 1909 as “the molecular structure that carries the essential characteristics (*phoros*) responsible for the biological activity of a drug (*pharmacon*)” [87]. In 1977, Peter Gund defined pharmacophore as “a set of structural characteristics of a molecule that is recognized by the receptor and that is responsible for the biological activity of the molecule” [88]. More recently, in 1998, the *International Union of Pure and Applied Chemistry* (IUPAC) defined pharmacophore as “an ensemble of steric and electronic features that is necessary to ensure optimal supramolecular interactions with a specific biological target and to trigger (or block) its biological response” [89,90]. Therefore, different molecules can act in the same target protein since they share the “same” pharmacophore (features) [91].

Pharmacophore modeling is a fast and efficient method that can be used, for example, as a filter to screen a virtual library of billions of compounds and identify molecules that share identical features to the ones present in the previously generated pharmacophore (and, hopefully, find new scaffolds for the target of interest). This makes this methodology a very important tool when we are trying to identify new hit compounds [72,91,92].

According to Pautasso et al. [93], pharmacophore models can be generated using two main approaches: (I) based on the structure of active molecules (ligand-based) and (II) based on the structure of the target (structure-based). 

The ligand-based pharmacophore modeling consists in the alignment of a set of active molecules to recognize common chemical features that are critical for their bioactivity. It is a possible and interesting alternative when sufficient information on active ligands is available and more importantly when no information about the 3D structure of the target (receptor) is accessible [72]. Pharmacophore generation from ligands may be obtained by two main approaches: shared feature pharmacophore (also known as common feature pharmacophore, in which a pair of similar conformations exists between different chemical structures that share the same chemical activities at a similar position) and quantitative SAR-based pharmacophore [93].

The structure-based pharmacophore modeling approach (also known as receptor-based) defines possible interaction points between the 3D structure of the macromolecular target or a macromolecule-ligand complex, which lead to the following subcategories: macromolecule-ligand complex based and macromolecule-based (without ligand) [93].

Several programs can be used to generate pharmacophore models including CATALYST (Accelrys) [94], Phase (Schrodinger) [95] and Molecular Operating Environment (MOE, Chemical Computing Group) [84], among others.

By analysis of the existing bibliography, two pharmacophore models were developed for the discovery of human proteasome inhibitors and are described below (Table 3).

In 2009, Lei et al. [96] developed a pharmacophore model (PM) for the design of proteasome inhibitors based on dipeptide inhibitors with boron atoms in their constitution (using a training set of 24 compounds). They obtained a model with seven features: two hydrogen bond acceptors, two hydrogen bond donors, one ionizable positive feature, and two ionizable hydrophobic features using CATALYST software (Table 3). This ligand based PM was validated using a test set of 26 molecules (including bortezomib) and allowing new lead compounds to be identified [96]. 

Li et al. [97] discovered in 2014 novel covalent proteasome inhibitors by using a combined strategy: pharmacophore modeling, molecular docking calculations, and molecular dynamics. The authors developed a structure-based PM using the murine constitutive 20S proteasome complexed with the epoxyketone inhibitor PR-957—the binding pocket of the human proteasome is similar to the murine one. LigandScout 3.03 software was used to detect the interactions and to generate the PM [97]. The best PM obtained contains two hydrogen-bond acceptors, two hydrogen-bond donors, one hydrophobic group, and several excluded volumes [97]. The validation of this PM was performed by using three known ligands and evaluated using ROC analysis (Table 3) [97].

### 2.3. Molecular Docking

Molecular docking was intensively used in the discovery and optimization of human proteasome inhibitors. Important insights on the proteasome-ligand interactions were revealed using this methodology.

Protein-ligand molecular docking is a computational method for predicting the energetically best pose of how a ligand interacts/binds in a protein binding pocket [66]. Protein-ligand docking mimics the recognition process in which a small molecule (ligand) translates, rotates, and twists exhaustively in the active site of a macromolecule (protein) with the goal of finding the energetically most favorable conformation binding mode (search algorithm), being the protein-ligand affinity (binding energy) estimated by a scoring function (scoring algorithm) [72,98].

Molecular docking can be classified into covalent and non-covalent docking depending if there is or not an implicit bond between the target and the ligand., Since the bulk of rational drug design relies on the discovery of noncovalent interactions, protein-ligand docking usually focuses on the docking between the target and the ligand through noncovalent interactions (hydrogen bonding, van der Waals, and electrostatic interactions), and in this case we call it noncovalent docking. However, if an implicit bond is established (via a link atom) between the target and the ligand during the docking calculations we call it covalent docking. Due to a growing interest to the design of covalently bonded inhibitors (e.g., irreversible inhibitors), several docking packages are now able to also perform this kind of calculation. GOLD software is one of these packages, able to perform noncovalent and covalent docking [99,100,101].

Molecular docking can also be classified as rigid (when ligand and protein structures are both treated as rigid entities; the ligand may only have translation and rotation movements inside the active site), semi-flexible (only the ligand structure has flexibility), and flexible (when the ligand and protein are considered flexible entities) [66,72,102]. The most common type of calculation is semi-flexible docking. However, receptor flexibility plays a key role in biomolecular recognition and is one of the important sources of errors in this type of calculation. Some docking software can now account for, at least, some flexibility of selected binding pocket residues [101,103]. 

At least three criteria can be considered to evaluate the accuracy of docking methods: the accuracy in the prediction of the ligand positioning (RMSD between docked and crystallographic poses should be ≤ 2 Å), the estimated free energy of the protein-ligand complex, and the ability to distinguish between active and inactive compounds [104]. To estimate the free energy of the protein-ligand complex, scoring functions are used. Traditional scoring functions include empirical, force-field, and knowledge based, however this issue continues to be a pitfall in docking calculations, despite the efforts in recent years [105,106]. Throughout a docking calculation it is important to have extensive information about the protein and the ligand to setup docking calculations to predict how the compounds interact with the receptor. First of all, it is critical to identify the location of the binding pocket in the target protein. However, we must not forget that the ligand may bind far from the classic binding site (allosteric binding). Once the binding site is identified, it should be characterized, for example, on its polarity, any charged areas, which residues are available to interact with the ligand(s), if some metal groups are needed for eventual interactions, and the size of the binding pocket [72]. Applications of molecular docking in the drug discovery process include the search for active ligands (hit compounds) for a specific protein from a virtual library of compounds (virtual screening), the optimization of hit compounds in order to obtain more potent molecules (optimized lead compounds) and even the identification of a protein that will specifically bind to a given compound, which can help to decipher the molecular mechanism of a certain drug when it is unknown [104].

Nowadays, several docking programs are available and a lot of variations have been implemented to accurately predict specific binding interactions. Some popular protein-ligand docking programs (namely GOLD [101] and AutoDock [107]) treat protein-ligand docking through an optimization procedure in which global optimization algorithms are used to optimize the poses obtained between a flexible ligand and a “rigid” protein target [98]. Other examples of docking programs include DOCK [108], FlexX [109], Surflex [110], GLIDE [111], Moldock [112], FRED [113] and ICM [102,105,114]. An extensive review on protein-ligand docking programs was recently published [115].

On recognizing the importance of achieving optimal and reliable molecular docking calculations, several efforts were made to compare the performance of different docking conditions, software, and protocols. However, comparing docking programs and protocols can be challenging. In fact, the performance can change considerably with the structure of the target protein, the selected docking protocol, the specific set of chosen variables/keywords, or even with the operator expertise [115]. 

In 2016, a very interesting, useful and updated benchmark exercise was published by Carlson et al. [116], in which they compared the performance of several docking calculations carried out by different groups using, for example, different protocols, software, and scoring functions, considering exactly the same three targets and the same ligands (donated by GSK). Some years before, using the Astex diverse set ([117]), Liebeschuetz et al. [118] compared the scoring functions implemented in GOLD software against this test set, identifying GOLD′s ChemPLP as the most effective scoring function for pose prediction. 

Molecular docking was by far the most used structure based computational approach for the discovery, design, and optimization of novel proteasome inhibitors using crystallographic protein structures of proteasomes from, e.g., *Bos tauros*, *Mus musculus*, *Saccharomyces cerevisiae*, and developed homology models as previously described. Different docking software (sometimes not the most popular one) and target structures were used for these calculations making difficult a direct comparison between them. However it is very interesting to see how this methodology has being applied and to analyze perspectives and improvements in the rational discovery of proteasome inhibitors.

In 2003, Kazi et al. [119] performed computational docking studies, using Autodock software and the crystal structure of the eukaryotic yeast 20S proteasome (PDB ID:1JD2; the yeast 20S proteasome is structurally similar to the mammalian 20S proteasome, being the CT-L active site of the two species highly conserved). Their study suggests that the interaction of genistein with the β5 subunit is responsible for inhibition of the CT-L proteasome activity. From these studies, it was possible to conclude that genistein places the A–C rings into the S1 pocket and that the hydroxyl group of the B-ring is close to Thr1 (1.85 Å), which may sterically block Thr1. Through an analogous protocol, in 2004 Smith et al. [120] observed that (−)-EGCG binds to the hydroxyl group of the N-terminal Thr of the proteasome′s CT-L active site in an orientation and conformation that is favorable for nucleophilic attack, resulting in inhibition of the proteasomal activity [120].

A series of vinyl sulfones synthesized by Rydzewski et al. [121] were further evaluated as inhibitors of the CT-like active site of the 20S proteasome. Docking calculations of these compounds were performed on the CT-L active site of the bovine proteasome crystallographic structure (PDB ID: 1IRU), using MOE software, and led to the discovery of novel and reversible 2-keto-1,3,4-oxadiazoles proteasome inhibitors that could work as key tool compounds for cellular and animal studies and further optimizations [121]. The same group [119,120,122,123] also demonstrated that the proteasome (especially the CT-L active site) is an important target of curcumin.

Yang et al. [124] found, using molecular docking calculations, that the proteasome is an important target of pristimerin in human prostate cancer cells, this inhibition being associated with apoptosis induction. The crystal structure of the eukaryotic yeast 20S proteasome was used for docking calculations (PDB ID: 1JD2; [119,120,122]) using AutoDock software. These studies revealed the interaction between pristimerin and the CT-L active site, suggesting that pristimerin interacts with the Thr1-N of the β5 subunit [124].

In 2008, Leban et al. [125] carried out studies to evaluate proteasome inhibition by peptide-semicarbazones. It was found that peptide-semicarbazones derived from Z-Trp-Trp-Phe-aldehyde inhibit the chymotryptic activity of the human proteasome in the nanomolar range but are less active against NF-κB. In contrast, cyclic semicarbazones, combine a strong inhibitory effect of the proteasome activity with an inhibition of NF-κB signaling in nanomolar concentrations [125]. The potential binding modes of the studied compounds were analyzed by refinement of docking poses generated by ProPose [126,127]. The crystallographic structure used for docking calculations was the PDB ID 1IRU (bovine 20S proteasome; resolution: 2.75 Å) and were selected the subunits that constitute the CT-L active site (β5 and β6 subunits) [125]. These docking studies showed that the 2-carbonyl of the hydantoin is likely to be coordinated to the N-terminal nitrogen of the catalytic Thr1 with the semicarbazone, coming close to the c-oxygen of Thr1 (3.4 Å). So, it is possible that the semicarbazone can be cleaved by the CT-L active site of the proteasome, this mechanism being a possible enzyme activated prodrug approach since the peptide aldehyde inhibitor is released from the semicarbazone (prodrug) only in the presence of the enzyme [125].

The peptide aldehyde inhibitor MG132 mode of action was evaluated by Zhang et al. [128], in 2009, combining covalent docking and molecular dynamics simulations. First of all, covalent docking studies (GOLD 4.0 software) in the CT-L active site (β5 and β6 subunits) were performed to generate the binding mode of MG132 to the 20S proteasome. Then two conformations with the lowest docking energy were selected for further binding mode analysis. The proposed model of the binding mode of MG132 to the proteasome showed a correlation between the structure and the activity of several proteasome inhibitors, especially at the P2 and P4 sites, being this information vital for the design of more potent proteasome inhibitors [128].

In 2010, Zhu et al. [129] synthesized, evaluated in vitro and in vivo, and performed docking calculations of a series of novel dipeptidyl boronic acid proteasome inhibitors composed by β-amino acids. Docking calculations were performed using the crystal structure with the PDB code 2F16, the protein and ligands being prepared with the software Insight II. GOLD 3.0 software was used for covalent docking calculations in the CT-L active site, applying a radius of 20 Å from the β5 catalytic N-terminal Thr1. Covalent docking was performed and the terminal boron atoms of all the ligands were bonded to the hydroxyl oxygen of Thr1 [129]. Docking calculations showed that the selected dipeptidyl boronic acid interacts with 20S proteasome in a similar way to the known proteasome inhibitor bortezomib [129,130]. Furthermore, pharmacokinetic profiles suggested that this dipeptidyl boronic acid has a bigger plasma exposure and a higher half-life than bortezomib [129].

In the same year, Kanwar et al. [131] performed a study on the potential interactions established between the proteasome β5 subunit and catechol-*O*-methyltransferase-resistant EGCG analogs. Molecular docking calculations were performed with AutoDock 3.0 software (Molecular Graphics Laboratory, The Scripps Research Institute, La Jolla, CA, USA), the target being the Thr1 of the β5 subunit (PDB ID: 1JD2– eukaryotic yeast 20S proteasome). The analogs docked in this study are structurally similar to (−)-EGCG and (−)-ECG, which suggested that these compounds may also act as proteasome inhibitors. The authors concluded that the major intermolecular interactions of these compounds come from hydrophobic interaction and hydrogen bonding. Highly susceptible structures have more hydrophobic interactions when compared to other structures, and with the hydrophobic amino acid residues in the β5-subunit of proteasome [131].

Also in 2010, Shi et al. [132] carried out docking simulations in order to identify the interaction mode of butyltin and phenyltin compounds (organotins) with proteasome β5 subunit (CT-L). Docking calculations were performed with AutoDock software by using eukaryotic yeast 20S proteasome. Computational and biological studies demonstrated different interactions with the β5 subunit. The authors concluded that tributyltin and triphenyltin were irreversible inhibitors, while organotins with one or two butyl substitutes or with one or two phenyl substitutes were reversible inhibitors [132].

In 2011, Bonfili et al. [133] identified and tested an EGCG oxidation derivative with proteasome modulatory activity. Besides a biological approach, molecular docking calculations were performed using InsightII software. Docking calculations were performed using the mammalian constitutive proteasome X-ray crystallographic structure (PDB ID: 1IRU-bovine); to obtain immunoproteasome catalytic subunits; homology modeling was performed using the PDB code 1IRU. The authors concluded that the EGCG oxidation derivative is biologically active towards isolated and cellular proteasomes, prolonging the efficacy of EGCG [133].

Also in 2011, Ma et al. [134] designed and synthesized a new series of peptide aldehyde derivatives, they being more active than the positive control MG132. A covalent docking protocol was followed to study the binding mode of these compounds. Docking calculations were performed in the crystal structure of yeast proteasome complexed with MG101, using GOLD 4.0 software. The analysis of the docking results showed that the most suitable length of the side chain in this Boc-series is Ser(OBzl) which leads to more active inhibition of the CT-L active site. When the residue at the P3 position is changed to proline, the results show that the pyrrolidine moiety projects into the S3 pocket, which makes the binding model of the main chain different from that of MG132, resulting in the disappearance of activity [134]. In conclusion, the docking mode observed is similar to the one present in the crystal complex and the P3-postion demonstrated to be crucial for the inhibitory potency [134].

In 2012, fourteen naphthoquinone derivatives, based on the non-peptidic proteasome inhibitor PI-083, were designed and tested by Xu et al. [135]. Six compounds demonstrated significant antiproliferative activities (IC_50_ values in the low micromolar range) and one of them was identified as a potent proteasome inhibitor by both in vitro and cell-based assays. A molecular docking study of the selected compound in the 20S proteasome CT-L active site suggests that this active naphtoquinone derivative has a binding mode in the CT-L active site similar to that of PI-083. Furthermore, docking calculations suggest that, compared to PI-083 (IC_50_ = 18.56 µM), the selected compound established two additional hydrogen bonding interactions with residues Thr1 and Gly23 of the 20S proteasome, which could explain its strong and increased inhibitory potency (3.65 µM) [135].

In the same year, the bovine 20S proteasome (PDB ID: 1IRU) was also used to perform docking calculations on the three proteasome catalytic sites by Pham et al. [136] who synthesized and characterized 29 cerpegin derivatives and evaluated them in the mammalian 20S proteasome. Computational and biological assays showed that these compounds were mainly selective for proteasome C-L activity, having IC_50_ values in the micromolar range, also suggesting a critical role of a Tyr residue (Tyr 114) belonging to the β2 subunit [136].

Santoro et al. [137] investigated the ability of porphyrins to inhibit the proteasome activity using one single molecule with different biological targets instead of a cocktail of active molecules. Besides biological studies, docking calculations were performed with cationic and anionic porphyrins into the β5 subunit of 20S proteasome complexed with bortezomib (PDB code: 2F16) using AutoDock Vina. The most active compound, H2T4, showed a similar inhibitory ability for all the three catalytic sites and was comparable with other inhibitors such as lactacystin (IC_50_ value in the µM range) [137].

Based on previous work developed by the same group (earlier mentioned as Pham et al. [136]), in 2013 Hovhannisyan et al. [138] performed in silico docking studies of three compounds, selected from C1 and N5 cerpegin derivatives, on the C-L active site using AutoDock software. The crystallographic structure used was the PDB code 1IRU (bovine proteasome). 

In 2013, Jiang et al. [139] performed docking studies on marchantin M, a noncompetitive inhibitor inhibitor of CT-L (IC_50_ = 6.99 µM) and C-L (IC_50_ = 5.33 µM) active sites of the 20S proteasome [139]. Docking calculations were performed with AutoDock Vina software. The target protein for docking was obtained from the yeast 20S proteasome crystal structure (PDB 2F16), being the size of the docking grid selected in order to cover the entire molecule [139].

In the same year, Orabi et al. [140] using Surflex-Dock program interfaced with SYBYL, performed the docking of syringic acid (a phenolic acid) and derivatives on the 20S proteasome (PDB ID:1R0P and 1JD2). Three-dimensional structure building and all modeling were performed using SYBYL (version X). Surflex-Dock version 2.0 (Tripos International, St. Louis, MO, USA) interfaced with SYBYL-X was used to dock TMC-95A, bortezomib and syringic acid derivatives in the active site of 20S yeast proteasome (PDB code: 2F16 and 1JD2) [140]. Docking of syringic acid derivatives revealed that the binding modes of energy-minimized derivatives are comparable to bortezomib bound conformation in the crystal structure of the eukaryotic yeast 20S proteasome (PDB code: 2F16) [140].

Also in 2013, computational studies of proteasome inhibition and apoptosis induction in human cancer cells by amino acid Schiff base-copper complexes were performed by Zuo et al. [141]. Software GLIDE from Schrödinger′s discovery Suite was used to perform docking calculations (PDB ID: 2F16-yeast 20S proteasome with bortezomib covalently bound), using bortezomib as control. Cellular and computational analyses of the results showed that two complexes containing 1,10-phenanthroline as the second ligand are potent proteasome inhibitors and apoptosis inducers in human cancer cells, while two complexes which involve 2,2′-bipyridine as the second ligand, presented no or little antineoplastic activity [141].

Bordessa et al. [142] synthesized in 2013 a library of pseudopeptides and studied their inhibitory activity in the rabbit 20S proteasome CT-L active site. Given the IC_50_ values obtained (0.7–85 µM), to find a possible explanation for the different activities of these compounds, molecular docking calculations were performed on the CT-L active site of the bovine proteasome crystallographic structure (PDB ID: 1IRU). Ligands were docked using GOLD 5.1 software (The Cambridge Crystallographic Data Centre, Cambridge, UK), the default parameters being considered. The scoring function selected was GoldScore and full torsional freedom was allowed for the side chains of Met45 and Ile35 (which belong to the S1 subpocket). Solutions were analyzed using Hermes 1.5 (The Cambridge Crystallographic Data Centre, Cambridge, UK). Giving the docking results, the estimated value of inhibition was determined according to its ability to reproduce the previous binding mode of noncovalent inhibitors considering three criteria: the first criterion being the tightness of the superimposition of its structure with that of the noncovalent pseudopeptidic inhibitor published by Furet et al. [85]; the second criterion considers the number of hydrogen bonds established with the key residues Thr1, Thr21, Gly23, Gly47, Ala49, and Asp125 (poses with more of these interactions were considered the best ones); the third criterion evaluates the ability of the ligand to occupy S1, S3, S4, and S5 specificity pockets of the 20S proteasome [142]. The docking results displayed hydrogen bond interactions with some, or all, key residues identified as being important for the binding of compounds in the 20S proteasome (Thr1, Thr21, Gly23, Gly47, Ala49, and Asp125) as well as the correct fitting of the specificity pockets S1, S3, and S4 or S5. The most active compound (IC_50_ value of 0.7 µM) established six hydrogen bonds with the target protein, out of which three involved key residues. Another potent compound also showed an IC_50_ value of 1.4 µM on the CT-L active site establishing four hydrogen bonds in total, out of which two were with the key residues Gly47 and Asp125. By superposing these two compounds within the CT-L active site, they became tightly aligned for most of their structure, a slight difference existing in the occupation of S5 instead of S4 [142]. In conclusion, Bordessa et al. proposed a binding mode standard for this class of noncovalent proteasome inhibitors of the CT-L active site [142]. 

In 2013, on knowing that the potency of covalent inhibitors can be significantly affected by their binding affinity for the target protein in the noncovalent binding mode, especially around its transition state to form covalent bonding, Kawamura et al. [143] proposed that, to design optimized covalent inhibitors, it is desirable to know the noncovalent binding mode of the lead inhibitor around the transition state. Kawamura et al. [143] studied the noncovalent binding mode of some covalent proteasome inhibitors (salinosporamide A derivatives) around the transition state by combined use of cyclopropylic strain-based conformational restriction and computational modeling (noncovalent docking with Glide 5.7 software in the CT-L active site of the PDB code 3GPT). The authors concluded that the combined use of a conformational restriction approach and docking simulations can be effective in investigating the noncovalent binding mode of covalent inhibitors around the transition state [143].

Later, in 2014, in order to analyze the binding mode of ridaifen-F (a nonpeptidic small-molecule proteasome inhibitor based on tamoxifen) derivatives of the proteasome, docking studies were performed [144]. The crystallographic structure of the yeast 20S proteasome complexed with fellutamide B (PDB ID: 3D29) was used for docking calculations of the ridaifen-F derivatives. Molecular Operating Environment version 2010.10 (MOE 2010.10) was the software selected for the calculations. The possible ligand-binding site was detected through application of Site Finder of MOE 2010.10, with the Connection Distance parameter set to 1.9 Å. Docking calculations were carried out using ASEDock. The output results suggested that the presence of two homopiperidine rings and the relationship between the homopiperidine rings and the side structures at the X position are important for the inhibition of the proteasome by ridaifen-F derivatives [144].

Also in 2014, Loizidou et al. [86] performed a computational study of the subunit specific interactions of the proteasome inhibitors argyrin A and F, with the aim of identify the determinants of subunit selectivity. Through the docking analysis of argyrin A at the active sites of the yeast proteasome, the conserved residues Thr1, Ser129, as well as the variable residues Gly168, Ser20, Thr21, Val31, Met45, and Ala49, were identified as interacting residues. Several of these residues are involved in specific interactions with argyrin A such as hydrogen bonding between Thr21 and a backbone amide at β1, β2, hydrogen bonding between Ala49 and a backbone amide at β1, β5, and hydrogen bonding of Gly47 and Ser96 at β5 [86].

Based on the optimization of previously described analogs of bortezomib bearing a bicyclic 1,6-naphthyridin-5(6*H*)-one scaffold as P3 fragment developed by the same group, in 2014 Troiano et al. [145] developed a new series of pseudopeptide boronate proteasomes with high target-selectivity and an optimal inhibition profile, since they inhibited two of the three proteolytic subunits of 20S proteasome, showing selectivity for the CT-L activity inhibition [145]. The biological evaluation of the compounds in the human 20S proteasome showed a promising inhibition profile, mainly for compounds bearing a P2 ethylene fragment (*K*_i_ values in the nM range for the CT-L active site). Docking experiments into the yeast 20S proteasome were performed according to a protocol previously applied by the same group [146] and showed that the ligands are accommodated mostly into the CT-L site, establishing a covalent bond with the catalytic Thr1 via the boron atom [145].

In 2014, Voss et al. [147] studied α-keto phenylamides as possible P1′-extended proteasome inhibitors. The aim of this study was to improve both potency and ligand efficiency of a compound previously discovered (lead structure) while avoiding significant enlargement (because too lipophilic and thus undruggable compounds may be originated) [147]. The SAR study and docking calculations identified a compound [tripeptidic α-keto phenylamide, IC_50_ = 38 nM − CT-L active site] as an improved derivative of an alpha-ketoamide, being a cell-permeable and slowly reversible covalent inhibitor which targets both the primed and non-prime sites of the binding pocket [147]. Covalent docking calculations of the ligands on the CT-L active site with flexible side chains of the receptor were performed in yeast 20S proteasome. In terms of preparation of the crystallographic structure, all unbound water molecules were removed, hydrogens were added and energy was minimized using the Amber12EHT force field. The pocket atoms of the receptor were tethered to allow movement. All other atoms were fixed. To estimate the binding free energy score, the scoring function used was London dG [147]. The S1′ occupation of the ligand as an additional selectivity criterion may diminish off-target side effects, such as peripheral neuropathy. Furthermore, the reversible binding mode of hemiketal formation is likely to enable penetration of deeper solid tissues and allow cells to recuperate unless they are sufficiently damaged. In conclusion, this new inhibitor is a promising drug candidate with antineoplastic properties [147]. 

In 2015, Scotti et al. [148] performed studies of a new series of peptide-based analogues which have a naphthoquinone pharmacophoric unit (2-chloronaphthoquinone) at the C-terminal position. These studies are based on the rationale that the proximity of the hydroxyl group of the catalytic Thr to the 2-chloronaphthoquinone unit is potentially able to attack carbon 2 of the pharmacophoric unit. Some naphthoquinone dipeptides showed proteasome inhibition in the µM range of the C-L and CT-L active sites of the 20S proteasome, namely two of these derivatives which were the most active derivatives against β1 and β5 subunits , presenting IC_50_ values of 0.73 µM for C-L and 39.40 µM for CT-L activity and IC_50_ values of 1.94 µM for C-L and 24.79 µM for CT-L activity [148]. The equilibrium geometry of the most active naphthoquinone dipeptide was obtained through semi-empirical PM3 calculations and subsequently used in docking calculations. The crystallographic structures used for docking calculations were yeast 20S proteasome complexed with epoxomicin (PDB ID: 1G65; resolution: 2.25 Å) and yeast 20S proteasome complexed with vibralactone (PDB ID: 4LQI, resolution: 2.7 Å) for C-L and CT-L active sites, respectively [148]. All the calculations were carried out using MOE-Dock integrated in the MOE software [148]. The docking studies suggested the same interaction for the compounds involved in this study, making reasonable the nucleophilic substitution of chlorine in position 2 [148]. The secondary interactions that stabilize the enzyme-inhibitor binding are most effective when the dipeptide sequence establishes features which are more hydrophobic. As suggested by docking calculations, the enzyme-inhibitors interaction should be non-covalent, this being related to reversible inhibition (confirmed with in vitro studies). The results of the docking calculations showed that the naphthoquinonic unit is near the Thr1 of the active site: on the C-L active site, the O_2_ quinonic oxygen is located near Thr1 and Gly47 residues at a distance of some 2.6–2.7 Å, which allows the formation of hydrogen bonds; on the CT-L active site, the O_1_ oxygen of the naphthoquinonic unit is at a higher distance from Thr1 (around 3.1 Å) but additional interactions occur between the functional groups of the N-terminal fragment and Ser115, Gln131, Gly128h and Asp116 residues at ligand-protein distances in the range 3.0–3.5 Å, contributing to the binding stability [148].

Based on the chemical templates of the known non-covalent 20S proteasome inhibitor TMC-95A and another selected compound, in 2015 Xu et al. [149] achieved the discovery of a novel series of noncovalent proteasome inhibitors through a fragment-based drug design approach. The compounds were synthesized and the inhibitory activity was evaluated against the CT-L active site, three of those compounds being potent inhibitors with IC_50_ values in the submicromolar range [149]. Docking calculations of the most potent compound (IC_50_ = 0.29 µM) were carried out with the Discovery Studio 2.5 software package (Accelrys, San Diego, CA, USA). The structure of the β5 and β6 subunits (CT-L active site) of the 20S proteasome, were obtained from the crystallographic structure with the PDB ID 3SDK. Docking calculations were performed with the Ligandfit protocol using the default settings. Maestro (version 9.3) was used to plot the ligand interactions. The tripeptide backbone established several hydrogen bonds with the active site residues (e.g., Thr21, Gly47, and Ala49 in the β5 subunit and Asp114 in the β6 subunit). The R1 and R2 side chains extended to the S3 and S1 pockets. Hydrogen bonds were detected between the hydroxyl group in R2 and Thr1 of the β5 subunit, as well as the carbonyl oxygen atom in R2 and Gly 47. Furthermore, Π-Π stacking and Π-cation interactions were also observed between Tyr96, His98 and the phenoxyphenyl moiety [149]. These studies provided a new chemical template for non-covalent proteasome inhibitors, being a good insight into future structural and optimization studies to improve potency and subunit selectivity [149].

### 2.4. Virtual Screening

Virtual screening (VS) is a very helpful technique to identify possible hit compounds from virtual libraries of billions of compounds during the drug development process. Later on, the selected compounds should be tested experimentally [66]. VS can follow either a ligand-based strategy (ligand-based virtual screening—LBVS) or a structure/target-based strategy (structure-based virtual screening—SBVS) [66,150]. LBVS techniques are based on the assumption that compounds with a similar topology have similar biological activity. So, a set of known active compounds is used as a starting point to build models (such as pharmacophores, as previously described), but may also use compounds of 2D and 3D similarity and substructure searching procedures. LBVS typically uses topology-based descriptors involving the pharmacophoric sites of the molecules. The descriptors of the known active molecules and the potential hit molecules are compared using pre-defined mathematical expressions to quantify molecular similarity. These approaches basically neglect any information about the target biomolecule as well as the 3D structure of the ligand compounds. Nevertheless, they are very efficient and are often applied in combination with other structure-based approaches to identify potential bioactive hits that can then be used in docking experiments [66,151]. SBVS usually employs molecular docking (described before) or target-based pharmacophores to search for compounds predicted to fit well and interact with the target protein binding pocket. This approach has the advantage of being able to incorporate information on the size and shape of the binding pocket in the form of excluded volumes [66]. The aim of this approach is the ranking of the screened compounds based on the prediction of binding modes and affinity between compounds and the target protein in order to identify molecules that better interact with the active site [152]. The combination of docking calculations with pharmacophores in VS procedures has proven to be really important in drug discovery and raises the efficiency of optimization of compounds, reducing possible problems resulting from insufficient consideration of protein flexibility or the use of inadequately optimized scoring functions [90].

In 2010, Basse et al. [153] discovered novel drug-like inhibitors of mammalian proteasome 20S through a multistep combining a structure-based and ligand screening approach using several docking softwares (FRED, LigandFit, Surflex). They performed several test cases in order to ensure that the docking-scoring combinations used in this study could reproduce known experimental structures of the proteasome co-crystallized with covalent compounds. They virtually screened the Chembridge compound collection (~300,000 molecules) after in silico ADME/Tox filtering. The crystallographic structure used to perform docking calculations has the PDB code 1IRU (from bovine proteasome), β5 and β6 subunits (chains L and M) being selected to form the structure of the CT-L active site. After docking/scoring and visual inspection, 200 molecules were selected for experimental testing against the CT-L activity of the rabbit 20S proteasome [153]. PA and T-L activities were also investigated using fluorogenic substrates. Cytotoxicity assays were performed with different cell lines (HeLa and HEK-293). A sulfanilic acid derivative N-acylated by a 4-substituted benzoyl group was demonstrated to be a promising compound and so it was redocked with Surflex and the energy was minimized in the binding pocket with MolDock. The authors concluded that by acting on one, two or three active site(s), the inhibitors may differentially reduce protein degradation and help to control normal cell cytotoxicity [153].

A few years later, in 2013, Maréchal et al. [154] used a structure and ligand-based in silico approach to identify commercially available 1,2,4-oxadiazole derivatives as non-covalent human 20S proteasome inhibitors. Docking calculations were performed over the CT-L active site of the constitutive 20S proteasome using both structure and ligand-based virtual screening calculations [154]. Structure-based virtual screening experiments were performed using the same procedure as described previously by Basse et al. [153]. They started by rigid docking of the ChemBridge compound collection with MS-Dock and flexible docking with LigandFit and Surflex. The top 100,000 molecules resulting from rigid-docking were re-docked using both LigandFit and Surflex. Then 300 molecules were purchased from the ChemBridge chemical vendor and tested experimentally. Optimization led to a compound that is a mixed proteasome inhibitor of the CT-L activity (*K*_i_ of 26.1 nM) [154].

Li et al. [97] performed virtual screening of SPECS database (~371,557 compounds) by using noncovalent docking and a PM based on the 20S proteasome, resulting in the selection of 2167 compounds. After molecular dynamics simulations, two hit compounds were selected [97].

Later, in 2015, Miller et al. [155] carried out the virtual screening of ∼340,000 small molecules against the CT-L active site of proteasome, followed by in vitro studies and structure optimization of the identified non-peptide, reversible proteasome inhibitor lead compound which has a pyrazole scaffold and targets β5 and β5i subunits, presenting good metabolic stability and effectiveness in suppressing solid tumor growth in vivo [155]. Structure-based virtual screening was based on a immunoproteasome model previously developed by the same group [156]—catalytic activities of the immunoproteasome subunits β2i and β5i are relatively similar to those present in the constitutive proteasome. After this 345,447 compounds included in the University of Cincinnati library were docked using FRED (OpenEye Scientific Software, Santa Fe, NM, USA) to perform rigid docking calculations. Based on consensus scoring, force field based energy scoring functions (MM-PBSA and MM-GBSA and manual visualization of binding modes), 288 compounds were selected for experimental validation. 19 of these 288 compounds were found to be active at 5 μM in a CT-L activity assay using the immunoproteasome. The structure−activity relationship (SAR) was studied by docking simulation of the previously mentioned lead compound on the β5 subunit, the results suggesting that the improved activity of the lead compound over the compound from which it derives may be promoted by hydrogen bonds created by the introduction of an amide linkage at ring A. Ring B is predicted to occupy the S3 specificity pocket. Ring D of the lead compound is predicted to occupy the S1 specificity pocket [155].

In 2015, Pundir et al. [157] preformed the screening of a chemical library constructed using a hybrid approach that incorporated a 4-piperazynilquinoline scaffold and a sulfonyl pharmacophore. Docking calculations were performed on the β subunits to investigate potential interaction sites, using crystallographic structures with the PDB ID 2F16 and 1IRU. The active site was searched using Induced-fit docking protocol with Site finder of Molecular Operating Environment (MOE) software. By this procedure, compound VR23 was identified as a small molecule capable of inhibiting the three proteasome catalytic sites in a nM/low µM range (the primary target of VR23 was β2 subunit). In combination with bortezomib, VR23 produced a synergistic effect in killing multiple myeloma cells. VR23 was effective in vivo to control multiple myeloma and metastatic breast cancer cells. It also enhanced the antitumor activity of paclitaxel, also reducing side effects. The authors concluded that VR23 is a novel proteasome inhibitor with good properties as an anticancer compound [157].

Several approaches and combined methodologies were used, namely to calculate binding energies and to elucidate the catalytic mechanism for proteasome inhibition.

A table compiling all the docking calculations in this overview is available as supporting information (Table S1).

### 2.5. Combined Methods: Docking, Quantum Mechanics, QM/MM and Molecular Dynamics

In 2012, Wei et al. [158] performed QM/MM-FE calculations to elucidate the detailed mechanism for the inhibition reaction of proteasome with the inhibitor epoxomicin. The selected structure of the enzymatic system in the reactant state was obtained from the crystallographic structure of yeast proteasome complexed with epoxomicin (PDB ID: 1G65), β5 and β6 subunits were selected (CT-L active site). The initial structure of the reaction system was energy-minimized with the MM method (AMBER11 software [158]. The results showed that the most favorable reaction pathway is associated with direct proton transfer, rather than water-assisted proton transfer, consisting of five reaction steps: first a direct proton (Hγ) transfer occurs from the Thr1-Oγ to the Thr1-Nz atom in order to activate the Thr1-Oγ. Next, the negatively charged Thr1-Oγ nuncleophilic atom attacks the carbonyl of epoxomicin. In the third step, the proton (Hγ) is transferred from the Thr1-Nz atom to the carbonyl oxygen of the inhibitor. The next step is a concerted process because the nucleophilic attack on the epoxomicin-C2 atom by the Thr1-Nz is coupled with the breaking of the C2−O2 bond in epoxomicin through a SN2 nucleophilic substitution. The fifth step includes a proton (Hz) transfer from the Thr1-Nz to the negatively charged O2 atom of the inhibitor [158]. The energy profile of the most favorable reaction pathway associated with the direct proton transference (without water molecules) was calculated and free energy barriers were obtained for the first, second, fourth, and fifth reaction steps: 9.9, 9.0, 23.6, and 1.2 kcal/mol, respectively. The value of the calculated free energy barrier calculated for the rate-determining step (the fourth step) is close to the experimentally derived activation free energy of ∼21−22 kcal/mol, which supports the performed calculations [158]. The proteasome-epoxomicin complex was neutralized by adding four chloride ions. Next, this system was solvated in an orthorhombic box of TIP3P water molecules (minimum solute-wall distance: 10 Å), the system being refined by a MD simulation (~48 ns) [158]. The enzyme reaction path was studied by using pseudo bond QM/MM calculations at the B3LYP/6-31G*:AMBER level: the QM calculations were performed at the B3LYP/6-31G* level of theory by using a modified version of Gaussian03 and MM calculations carried out through a modified version of the AMBER8 software [158]. After the calculation of the minimum-energy path by QM/ MM, the free energy changes associated with the QM/MM interactions were determined by using FEP (the used time step was 2 fs, bond lengths involving hydrogen atoms were constrained) [158]. 

A few years later, in 2015, another study also performed by Wei et al. [159] elucidated the detailed mechanism of the inhibition of the proteasome by the inhibitor syringolin A (SylA) through QM/MM-FE calculations [159]. The crystallographic structure used was the yeast proteasome-SylA complex (PDB ID: 2ZCY) and β5 and β6 subunits were selected to perform the calculations [159]. The results showed that the reaction pathway has three steps: first, a direct proton transfer occurs from the Thr1-Oγ to the Thr1-Nz to activate the Thr1-Oγ; in the second step (which seems to be the rate-determining step) the negatively charged Thr1-Oγ (nucleophile) attacks the olefin carbon of SylA; the final step consists of the migration of the proton (Hγ) from the Thr1-Nz to the negatively charged C2 atom of SylA. The free energy barriers for the first and second reaction steps are 9.8 and 17.3 kcal mol^−1^, while the third step is barrier less [159]. The results also showed that no water molecules can assist the rate-determining step (i.e., the second step), since this reaction step does not involve a proton transfer. The calculated free energy barrier of 24.6 kcal mol^−1^ for the rate-determining step is similar to the experimental results (∼22.4–23.0 kcal mol^−1^), which supports the calculations performed. Additionally, the results obtained showed that the reverse reaction is extremely slow, the nucleophilic attack of Thr1-Oγ to the olefin being irreversible [159].

Sun et al. [160] performed the optimization of furan-based peptides through the design and synthesis of a series of dipeptidic and tripeptidic inhibitors with the aim of improving their potency and solubility. In vitro and in silico studies were also performed. Most of the tripeptidic inhibitors demonstrated enhanced potency and selectivity on the CT-L active site in both enzymatic and cellular assays, as well as good antineoplastic activities in various tumor cell lines. However, dipeptidic compounds showed no inhibitory effects, leading to the assumption that a noncovalent binding mode is adopted. Molecular docking studies and molecular dynamics simulations were carried out to verify this hypothesis. It was observed that the distance between the furyl ketone motif and the catalytic Thr1 is too long (4.9 Å) to form a covalent bond. The crystallographic structure of the target protein selected has the PDB ID 4NO8 (20S yeast proteasome), β5 and β6 subunits were selected. Before docking calculations, the protein and ligands were prepared with Discovery Studio 2.5 software: correct protonation at pH 7.4, charges calculated, water removed, and energy minimized. AutoDock 4.2 was the software selected to perform docking calculations of the ligands, the default parameters being used, except for those changes mentioned. The energy-scoring grid was prepared as a 40 × 40 × 40 Å box with a spacing of 0.375, centered on the ligand value (11.667, −137.254, 19.568) [160]. To obtain a more integrated and precise view of the binding process, molecular dynamics simulations were carried out with AMBER 11 software by starting from the docking pose of the most potent tripeptide derivative in complex with the CT-L active site of the 20S proteasome [160]. Calculation of RMSDs with respect to the starting structure confirmed the stability of the trajectory. A more stable and reasonable binding mode for the most potent tripeptide derivative with the protein was obtained and seven hydrogen bonds with Arg19, Thr21 (2), Ala49, Ser130, Glu132, and Arg137 were established [160]. In conclusion, computational studies suggested a noncovalent binding mode which can be very useful for future structural modifications and synthesis of more potent and selective proteasome inhibitors [160].

## 3. Conclusions

Proteasome is nowadays an established key target in pharmacological research. The last two decades were prolific for the discovery and optimization of novel proteasome inhibitors, some of them are now already in clinics. As an example, Bortezomib (Velcade), the first marketed proteasome inhibitor, greatly contributed over the last decade to improvements in the prognosis of patients with multiple myeloma. Development of chemoresistance against proteasome inhibitors, its severe side effects, and its inefficacy against several solid tumors emphasized the need for the development of better drugs. The application of computer-aided drug design methodologies was crucial to assist in the efforts to improve and speed up the discovery of compounds that show a significant inhibitory activity against human proteasome. In this overview the application of several in silico methodologies, such as homology modeling, pharmacophore generation, molecular docking, virtual screening, quantum mechanics, and molecular dynamics to identify and optimize new proteasome inhibitors as well as to give invaluable insights on the key interactions and catalytic mechanisms involved in proteasome inhibition, was revised. Before the availability of the human proteasome crystallographic structure, several homology models were created to be used in structure based methodologies like molecular docking. Molecular docking calculations were extensively performed to rationalize the importance of key interaction inside the proteasome binding pocket, to establish structure-activity relationships and to drive synthetic efforts. Pharmacophore models were successfully generated and allow the identification of essential features present in proteasome inhibitors. Based on previous studies, structure based and ligand based virtual screening campaigns carried out against chembridge, specs, and other databases were able to identify novel and chemically diverse proteasome inhibitors. In addition, some in silico methodologies were synergistically combined to explain and predict proteasome modulation. We believe that, as the crystallographic structure of human proteasome is now available, the progress in the discovery of new compounds could be greatly improved and become successful. In conclusion, the efforts concerning the development of proteasome inhibitors from a computational point of view were presented here and discussed, providing an overview of the current state-of-the-art that we hope can guide further developments in this field. 

## Figures and Tables

**Table 1:** Representative examples of proteasome inhibitors and IC_50_ values on the targeted active site(s).

Proteasome Inhibitors	Structural Class	Catalytic Subunit			
		β1	β2	β5	Reference
Bortezomib	Boronates	74 nM	4200 nM	7 nM	[49]
Carfilzomib	Epoxyketones	2400 nM	3600 nM	6 nM	[49]
Delanzomib	Boronates	<100 nM	>100 nM	3.8 nM	[50]
Epoxomicin	Epoxyketones	−	−	5.7 nM	[51]
Fellutamide B	Aldehydes	1200 nM	2000 nM	9.4 nM	[51]
Ixazomib	Boronates	31 nM	3500 nM	3.4 nM	[52]
Marizomib	β-Lactones	330 nM	26 nM	2.5 nM	[53]
MG-132	Aldehydes	1400 nM	4500 nM	68 nM	[54]
Oprozomib	Epoxyketones	−	−	36 nM	[55]

**Table 2:** Examples of PDB ID of different organisms and identity percentage when compared to the human proteasome.

Organism	PDB ID (Resolution)	Percentage Identity		
		β1	β2	β5
***Homo sapiens***	4R3O (2.60 Å)	−	−	−
	4R67 (2.89 Å)			
***Bos taurus***	1IRU (2.75 Å)	94	99	96
***Mus musculus***	3UNB (2.90 Å)	94	97	95
	3UNE (3.20 Å)			
***Saccharomyces cerevisia***	3GPT (2.41 Å)	55	19	67
	5CZ4 (2.30 Å)			
	4NNN (2.50 Å)			
	3D29 (2.60 Å)			
	3MG0 (2.68 Å)			
	3UN8 (2.70 Å)			
	4INR (2.70 Å)			
	2F16 (2.80 Å)			
	1JD2 (3.00 Å)			

**Table 3:** Pharmacophore models applied for the discovery of human proteasome inhibitors.

Reference	Software	Training Set	Test Set	PDB	PM Features
Lei et al. [96]	Catalyst	24 dipeptide inhibitors	26 molecules	−	2 HBA ^1^, 2HBD ^2^, 2 Hyd ^3^, 2 PI ^4^
Li et al. [97]	LigandScout	−	3 molecules	3 UNB	2 HBA ^1^, 2 HBD ^2^, 1 Hyd ^3^, several excluded volumes

^1^ Hydrogen bond acceptor, ^2^ Hydrogen bond donor, ^3^ Ionizable hydrophobic feature, ^4^ Ionizable positive feature.

## Supplementary Materials

Supplementary materials can be accessed at: http://www.mdpi.com/1420-3049/21/7/927/s1.
