# Supplementary material for: Computational Approaches for the Discovery of Human Proteasome Inhibitors: An Overview
Source: Molecules. 2016 Jul 16;21(7):927. doi: 10.3390/molecules21070927 (PMC6274525; doi:10.3390/molecules21070927)
Supplement: Supplementary file 1 [file molecules-21-00927-s001.pdf]

Supplementary Materials: Computational Approaches for the Discovery of Human Proteasome Inhibitors: An Overview

Romina A. Guedes [1], Patrícia Serra [1], Jorge A. R. Salvador [2,3] and Rita C. Guedes [1,*]

Table S1. Computational studies on the field of proteasome inhibitors.

Reference	Software	PDB ID	Active site	Database/compound(s) studied
Kazi *et al.* [1]	Autodock	1JD2	β5	Genistein
Smith *et al.* [2]	Autodock	1JD2	β5	(-)-EGCG
Rydzewski *et al.* [3]	MOE	1IRU	β5	Vinyl sulfones
Yang *et al.* [4]	Autodock	1JD2	β5	Pristimerin
Milacic *et al.* [5]	Autodock	1JD2	β5	Curcumin
Leban *et al.* [6]	ProPose	1IRU	β5	Peptide-semicarbazones
Zhang *et al.* [7]	GOLD	Crystal structures of MG101-proteasome complex		MG132
	MD: Amber (Divcon and Antechamber modules – determination of partial atomic charges)			
Basse *et al.* [8]	FRED, LigandFit, Surflex MolDock	1IRU	β5	Chembridge compound collection (~300000 molecules)
Zhu *et al* [9]	GOLD	2F16	β5	Dipeptidyl boronic acids
Kanwar *et al.* [10] (Smith *et al.* [2])	AutoDock	1JD2	β5	Catechol-*O*-methyltransferase-resistant EGCG analogs
Shi *et al.* [11]	AutoDock	Eukaryotic yeast proteasome	β5	Organotin compounds (butyltins and phenyltins)
Bonfili *et al.* [12]	InsightII	1IRU		EGCG oxidation derivative
Ma *et al.* [13]	GOLD	Yeast proteasome:MG101 complex	β5	Peptide aldehyde derivatives

Table S1. *Cont.*

Reference	Software	PDB ID	Active site	Database/compound(s) studied
Xu *et al.* [14]			β5	Naphthoquinone derivatives
Pham *et al.* [15]		1IRU	β1, β2 and β5	Cerpegin derivatives
Santoro *et al.* [16]	AutoDock Vina	2F16	β5	Cationic and anionic porphyrins
Hovhannisyan *et al.* [17]	AutoDock	1IRU	β5	Cerpegin derivatives
Maréchal *et al.* [18]	MS-Dock, LigandFit and Surflex.	Constitutive 20S proteasome	β5	ChemBridge compound collection
(same procedure as Basse *et al.* [8])				
Jiang *et al.* [19]	AutoDock Vina	2F16	β1, β2 and β5	Marchantin M
Orabi *et al.* [20]	Surflex-Dock interfaced with SYBYL-X	1R0P and 1JD2	β1, β2 and β5	TMC-95A, bortezomib and syringic acid derivatives
		2F16 and 1JD2		
Zuo *et al.* [21]	GLIDE	2F16		Amino acid Schiff basecopper complexes; bortezomib as control
Bordessa *et al.* [22]	GOLD	1IRU	β5	Library of pseudopeptides
Kawamura *et al.* [23]	Glide	3GPT	β5	Salinosporamide A derivatives
Li *et al.* [24]	Ph4 SB	3UNB	β5	Epoxyketones
	LigandScout			
	Noncovalent docking: LibDock	3UNB		
	Covalent docking: GOLD	3UNB		
	MD: Desmond software	3UNB		
	ADME: QikProp	—		
Hasegawa *et al.* [25]	MD: Chem3D		β1, β2 and β5	Tamoxifen derivatives (RID-A – RID-H)
	MOE	3D29		Ridaifen-F derivatives
	ASEDock			
Scarbaci *et al.* [26]	GOLD	2F16	β5	Peptidomimetic boronates

Table S1. *Cont.*

Reference	Software	PDB ID	Active site	Database/compound(s) studied
Troiano et. al. [27]	GOLD	2F16	β5	Pseudopeptide boronates
Voss *et al.* [28]	MOE	4R02	β5	α-Keto phenylamides
Miller *et al.* [29]	FRED		β5	345 447 compounds included in the University of Cincinnati library
			β5	Non-peptide, reversible proteasome inhibitor with a pyrazole scaffold
	MM-PBSA and MM-GBSA			
Scotti *et al.* [30]	MD: semi-empirical PM3 calculations			Naphthoquinone dipeptide derivatives
	MOE	1G65 (for β1) 4LQI (for β5)	β1	Naphthoquinone dipeptide derivatives
			β5	
Xu *et al.* [31]	Discovery Studio, Ligandfit protocol,	3SDK	β5	Linear peptide
Pundir *et al.* [32]	MOE	2F16 and 1IRU	β1, β2 and β5	4-piperazynilquinoline scaffold and a sulfonyl pharmacophore
Sun *et al.* [33]	AutoDock	4NO8	β5	Furan-based peptides (dipeptidic and tripeptidic inhibitors)
	MD: Sander module (AMBER 11) Ab initio: Gaussian	4NO8	β5	Tripeptide derivative (furan-based)

Reference:

1. Kazi, A.; Daniel, K.G.; Smith, D.M.; Kumar, N.B.; Dou, Q.P. Inhibition of the proteasome activity, a novel mechanism associated with the tumor cell apoptosis-inducing ability of genistein. *Biochem. Pharmacol.* **2003**, *66*, 965–976.
2. Smith, D.M.; Daniel, K.G.; Wang, Z.; Guida, W.C.; Chan, T.-H.; Dou, Q.P. Docking studies and model development of tea polyphenol proteasome inhibitors: applications to rational drug design. *Proteins* **2004**, *54*, 58–70.
3. Rydzewski, R.M.; Burrill, L.; Mendonca, R.; Palmer, J.T.; Rice, M.; Tahilramani, R.; Bass, K.E.; Leung, L.; Gjerstad, E.; Janc, J.W.; Pan, L. Optimization of subsite binding to the beta5 subunit of the human 20S proteasome using vinyl sulfones and 2-keto-1,3,4-oxadiazoles: Syntheses and cellular properties of potent, selective proteasome inhibitors. *J. Med. Chem.* **2006**, *49*, 2953–2968.
4. Yang, H.; Landis-Piwowar, K.R.; Lu, D.; Yuan, P.; Li, L.; Prem-Veer Reddy, G.; Yuan, X.; Dou, Q.P. Pristimerin induces apoptosis by targeting the proteasome in prostate cancer cells. *J. Cell. Biochem.* **2008**, *103*, 234–244.
5. Milacic, V.; Banerjee, S.; Landis-Piwowar, K.R.; Sarkar, F.H.; Majumdar, A.P.N.; Dou, Q.P. Curcumin inhibits the proteasome activity in human colon cancer cells *in vitro* and *in vivo*. *Cancer Res.* **2008**, *68*, 7283–7292.
6. Leban, J.; Blisse, M.; Krauss, B.; Rath, S.; Baumgartner, R.; Seifert, M.H.J. Proteasome inhibition by peptide-semicarbazones. *Bioorganic Med. Chem.* **2008**, *16*, 4579–4588.
7. Zhang, S.; Shi, Y.; Jin, H.; Liu, Z.; Zhang, L.; Zhang, L. Covalent complexes of proteasome model with peptide aldehyde inhibitors MG132 and MG101: Docking and molecular dynamics study. *J. Mol. Model.* **2009**, *15*, 1481–1490.
8. Basse, N.; Montes, M.; Maréchal, X.; Qin, L.; Bouvier-Durand, M.; Genin, E.; Vidal, J.; Villoutreix, B.O.; Reboud-Ravaux, M. Novel organic proteasome inhibitors identified by virtual and *in vitro* screening. *J. Med. Chem.* **2010**, *53*, 509–513.
9. Zhu, Y.; Zhu, X.; Wu, G.; Ma, Y.; Li, Y.; Zhao, X.; Yuan, Y.; Yang, J.; Yu, S.; Shao, F.; et al. Synthesis, *in vitro* and *in vivo* biological evaluation, docking studies, and structure-activity relationship (SAR) discussion of dipeptidyl boronic acid proteasome inhibitors composed of β-amino acids. *J. Med. Chem.* **2010**, *53*, 1990–1999.
10. Kanwar, J.; Imthiyaz Moahammad; Huanjie Yang; Huo, C.; Chan, T.H.; Dou, Q.P. Computational modeling of the potential interactions of the proteasome β5 subunit and catechol-*O*-methyltransferase-resistant EGCG analogs. *Int. J. Mol. Med.* **2010**, *26*, 837–843.
11. Shi, G.; Sun, Q.; Yang, H.; Dou, Q.; Deng, Q.; Wang, H.; Zhong, G. Molecular modeling for the interaction between proteasome beta 5 subunit and organotin compounds. *Sci. China Chem.* **2010**, *53*, 2387–2393.
12. Bonfili, L.; Cuccioloni, M.; Mozzicafreddo, M.; Cecarini, V.; Angeletti, M.; Eleuteri, A.M. Identification of an EGCG oxidation derivative with proteasome modulatory activity. *Biochimie* **2011**, *93*, 931–940.
13. Ma, Y.; Xu, B.; Fang, Y.; Yang, Z.; Cui, J.; Zhang, L.; Zhang, L. Synthesis and SAR study of novel peptide aldehydes as inhibitors of 20S proteasome. *Molecules* **2011**, *16*, 7551–7564.
14. Xu, K.; Xiao, Z.; Tang, Y.B.; Huang, L.; Chen, C.-H.; Ohkoshi, E.; Lee, K.-H. Design and synthesis of naphthoquinone derivatives as antiproliferative agents and 20S proteasome inhibitors. *Bioorg. Med. Chem. Lett.* **2012**, *22*, 2772–2774.
15. Pham, T.H.; Hovhannisyan, A.; Bouvier, D.; Tian, L.; Reboud-Ravaux, M.; Melikyan, G.; Bouvier-Durand, M. A new series of N5 derivatives of the 1,1,5-trimethyl furo[3,4-c]pyridine-3,4-dione (cerpegin) selectively inhibits the post-acid activity of mammalian 20S proteasomes. *Bioorg. Med. Chem. Lett.* **2012**, *22*, 3822–3827.
16. Santoro, A.M.; Lo Giudice, M.C.; D'Urso, A.; Lauceri, R.; Purrello, R.; Milardi, D. Cationic porphyrins are reversible proteasome inhibitors. *J. Am. Chem. Soc.* **2012**, *134*, 10451–10457.
17. Hovhannisyan, A.; Pham, T.H.; Bouvier, D.; Qin, L.; Melikyan, G.; Reboud-Ravaux, M.; Bouvier-Durand, M. C1 and N5 derivatives of cerpegin: Synthesis of a new series based on structure-activity relationships to optimize their inhibitory effect on 20S proteasome. *Bioorganic Med. Chem. Lett.* **2013**, *23*, 2696–2703.

18. Maréchal, X.; Genin, E.; Qin, L.; Sperandio, O.; Montes, M.; Basse, N.; Richy, N.; Miteva, M.A; Vidal, J.; Villoutreix, B.O. 1,2,4-Oxadiazoles identified by virtual screening and their non-covalent inhibition of the human 20S Proteasome. **2013**, doi:10.2174/0929867311320180006.
19. Jiang, H.; Sun, J.; Xu, Q.; Liu, Y.; Wei, J.; Young, C.Y.F.; Yuan, H.; Lou, H. Marchantin M: a novel inhibitor of proteasome induces autophagic cell death in prostate cancer cells. *Cell Death Dis.* **2013**, doi:10.1038/cddis.2013.285.
20. Orabi, K.Y.; Abaza, M.S.; El Sayed, K.A; Elnagar, A.Y.; Al-Attiyah, R.; Guleri, R.P. Selective growth inhibition of human malignant melanoma cells by syringic acid-derived proteasome inhibitors. *Cancer Cell Int.* **2013**, doi**:**10.1186/1475-2867-13-82.
21. Zuo, J.; Caifeng, B.; Yuhua, F.; Daniela, B.; Chiara, N.; Kenyon, G.D.; Ping, D.Q. Cellular and computational studies of proteasome inhibition and apoptosis induction in human cancer cells by amino acid Schiff base-copper complexes. *J. Inorg. Biochem.* **2013**, *118*, 83–93.
22. Bordessa, A.; Keita, M.; Maréchal, X.; Formicola, L.; Lagarde, N.; Rodrigo, J.; Bernadat, G.; Bauvais, C.; Soulier, J.-L.; Dufau, L.; et al. α- and β-hydrazino acid-based pseudopeptides inhibit the chymotrypsin-like activity of the eukaryotic 20S proteasome. *Eur. J. Med. Chem.* **2013**, *70*, 505–524.
23. Kawamura, S.; Unno, Y.; Tanaka, M.; Sasaki, T.; Yamano, A.; Hirokawa, T.; Kameda, T.; Asai, A.; Arisawa, M.; Shuto, S. Investigation of the noncovalent binding mode of covalent proteasome inhibitors around the transition state by combined use of cyclopropylic strain-based conformational restriction and computational modeling. *J. Med. Chem.* **2013**, doi:10.1021/jm400542h.
24. Li, A.; Sun, H.; Du, L.; Wu, X.; Cao, J.; You, Q.; Li, Y. Discovery of novel covalent proteasome inhibitors through a combination of pharmacophore screening, covalent docking, and molecular dynamics simulations. *J. Mol. Model.* **2014**, doi:10.1007/s00894-014-2515-y.
25. Hasegawa, M.; Yasuda, Y.; Tanaka, M.; Nakata, K.; Umeda, E.; Wang, Y.; Watanabe, C.; Uetake, S.; Kunoh, T.; Shionyu, M.; et al. A novel tamoxifen derivative, ridaifen-F, is a nonpeptidic small-molecule proteasome inhibitor. *Eur. J. Med. Chem.* **2014**, *71*, 290–305.
26. Scarbaci, K.; Troiano, V.; Ettari, R.; Pinto, A.; Micale, N.; di Giovanni, C.; Cerchia, C.; Schirmeister, T.; Novellino, E.; Lavecchia, A.; et al. Development of novel selective peptidomimetics containing a boronic acid moiety, targeting the 20s proteasome as anticancer agents. *ChemMedChem* **2014**, *9*, 1801–1816.
27. Troiano, V.; Scarbaci, K.; Ettari, R.; Micale, N.; Cerchia, C.; Pinto, A.; Schirmeister, T.; Novellino, E.; Grasso, S.; Lavecchia, A.; Zappalà, M. Optimization of peptidomimetic boronates bearing a P3 bicyclic scaffold as proteasome inhibitors. *Eur. J. Med. Chem.* **2014**, doi:10.1016/j.ejmech.2014.06.017.
28. Voss, C.; Scholz, C.; Knorr, S.; Beck, P.; Stein, M.L.; Zall, A.; Kuckelkorn, U.; Kloetzel, P.M.; Groll, M.; Hamacher, K.; Schmidt, B. α-Keto phenylamides as P1′-extended proteasome inhibitors. *ChemMedChem* **2014**, 2557–2564.
29. Miller, Z.; Kim, K.-S.; Lee, D.-M.; Kasam, V.; Baek, S.E.; Lee, K.H.; Zhang, Y.-Y.; Ao, L.; Carmony, K.; Lee, N.-R.; et al. Proteasome inhibitors with pyrazole scaffolds from structure-based virtual screening. *J. Med. Chem.* **2015**, doi:10.1021/jm501344n.
30. Scotti, A.; Trapella, C.; Ferretti, V.; Gallerani, E.; Gavioli, R.; Marastoni, M. Studies of C-terminal naphthoquinone dipeptides as 20S proteasome inhibitors. *J. Enzyme Inhib. Med. Chem.* **2015**, doi:10.3109/14756366.2015.1037749.
31. Xu, K.; Wang, K.; Yang, Y.; Yan, D.-A.; Huang, L.; Chen, C.-H.; Xiao, Z. Discovery of novel non-covalent inhibitors selective to the β5-subunit of the human 20S proteasome. *Eur. J. Med. Chem.* **2015**, *98*, 61–68.
32. Pundir, S.; Vu, H.-Y.; Solomon, V.R.; McClure, R.; Lee, H. VR23: A quinoline-sulfonyl hybrid proteasome inhibitor that selectively kills cancer via cyclin E-mediated centrosome amplification. *Cancer Res.* **2015**, 75, 7164-7175.
33. Sun, Q.; Xu, B.; Niu, Y.; Xu, F.; Liang, L.; Wang, C.; Yu, J.; Yan, G.; Wang, W.; Jin, H.; et al. Synthesis, bioactivity, docking and molecular dynamics studies of furan-based peptides as 20S proteasome inhibitors. *ChemMedChem* **2015**, *10*, 498–510.
